# An extended refutation text and funny videos reduce notorious *p*-value misconceptions

**DOI:** 10.1038/s41598-026-53375-w

**Published:** 2026-05-23

**Authors:** Markus H. Hefter

**Affiliations:** Department of Psychology, Bielefeld University, 33615 Bielefeld, Germany

**Keywords:** Mathematics and computing, Psychology, Psychology

## Abstract

For many decades, the vast majority of psychology students and their supervisors alike have been prone to misunderstanding the *p*-value, such as confusing it with a hypothesis’ probability. The present paper offers three innovative contributions to address this problem: (1) A novel yet valid methodological approach to assess *p-*value misconceptions that combines correctness and subjective certainty. (2) An effective short-term digital intervention built around an extended refutation text to reduce those misconceptions in about 10 min. (3) An unorthodox humor intervention with funny videos for extra effectiveness (and chuckles). A sample of 157 undergraduate psychology students (*M*_age_ = 23.36) took part in an online experiment featuring a 2×2-factorial design (funny vs. neutral videos and intervention vs. control condition. Planned contrast analyses revealed a large positive effect of the digital intervention on the participants’ conceptual change score. Furthermore, the prior presentation of funny videos did indeed yield an additional positive effect. Overall, the findings demonstrate the potential of extended refutation texts (enhanced with funny videos) for a short and convenient digital refresher aimed at psychology students who had already taken an introductory course but needed further clarification to tackle *p-*value misconceptions.

**Supplementary Information:**

The online version contains supplementary material available at 10.1038/s41598-026-53375-w.

## Introduction

What does a statistical test result that is significant at the 5% level actually mean? For instance, when a basic independent means *t-*test yields a *p-*value of .03? Of course, a *p-*value is simply the probability of the observed (or more extreme) data, given that the null hypothesis H_0_ is true^1^. For many decades however, the vast majority of psychology students and their supervisors alike have been prone to misunderstanding the *p-*value. For instance, they confuse it with the probability of the null hypothesis H_0_ or with being able to deduce the probability of the experimental hypothesis H_1_^2–8^. Have all the eager efforts of lecturers giving traditional statistics courses been in vain?

The present paper aims to address this problem by offering three innovations in the field of teaching and learning research methods. First, it proposes a novel yet valid methodological approach to assess *p-*value misconceptions by combining correctness and subjective certainty. Second, it presents an effective digital intervention built around an extended refutation text to reduce those misconceptions in about 10 min. Third and finally, it demonstrates an unorthodox humor intervention with funny video clips for extra effectiveness (and chuckles).

### The notorious *p*-value misinterpretations and their assessment

The vast majority of psychology students and their supervisors struggle with a basic understanding of *p-*values^3–7^. To assess *p-*value misinterpretations, studies haven usually relied on a six-item questionnaire by Oakes^7^ or modifications thereof. This questionnaire comprises a short introductory stem with a simple *t-*test output, such as “*t*(18) = 2.7, *p* = .01”, followed by six wrong statements about the output’s interpretation, such as “You have found the probability of the null hypothesis being true.” Participants are told to mark each statement as either true or false.

In Oakes’^7^ original study with this questionnaire, 97% of the 70 participating academic psychologists found at least one of the six misinterpretations of *p*value to be true. Falk and Greenbaum^4^ used a modification of Oakes’^7^ questionnaire, added the correct alternative (“none of the statements is correct”), and gave their learners a critical article to read^8^. Still, 87% of the 53 participating students agreed with at least one misinterpretation. For another instance, Haller and Kraus^3^ asked 113 psychology department members at six German universities to fill out the modified six-item questionnaire by Oakes^7^. The result thereof: 80% of the methodology instructors, 90% of the scientific psychologists and 100% of the psychology students, agreed with at least one of the six misinterpretations of *p-*values.

However, this method of letting participants grade given statements as either true or false does not allow differentiating between missing concepts and misconceptions. A student, who endorses a wrong statement (e.g., that a *p*-value enables one to deduce a hypothesis’ probability), might do so because of simply guessing due to missing concepts, or might do so because of actually believing the misconception. The difference lies in the subjective certainty. In other words, an incorrect answer could originate from both misconceptions and missing conceptions, but only a misconception would also feature high subjective certainty.

Eitel et al.^9^ in fact took subjective certainty into account when they developed their *Misconceptions about Multimedia Learning Questionnaire* (MMLQ). It addressed misconceptions covering the topics learning styles, hemispheric isolation, naïve summation, and motivation primacy. For each item, Eitel et al.^9^ multiplied the value for correctness (i.e., +1 or −1) by the subjective certainty rating from 0 (*very uncertain*) to 4 (*very certain*) resulting in a possible range from −4 to +4. This enables a convenient interpretation: High negative values (i.e., incorrect answer and high certainty) represent genuine misconceptions, high positive values (i.e., correct answer and high certainty) represent genuine correct concepts, whereas values around zero represented missing concepts (i.e., guessed answer and low certainty).

The present study builds on this method when assessing those *p-*value misinterpretations by combining correctness and subjective certainty as well.

### Extended refutation texts against *p-*value misconceptions

There are indeed effective long-term interventions to teach students basic concepts about null hypothesis testing and *p*-values, such as an 8-week MOOC^10^. For another instance, Reaburn^11^ relied on a one-semester unit and implemented instructional techniques such as computer simulations and guided discovery learning. Murdoch et al.^12^ also suggest implementing Monte Carlo simulations into statistic courses to demonstrate valid interpretations of *p-*values.

However, the present paper’s aim is to develop and test a very short-term digital intervention below the 15-minute mark. Its intended use is thus by no means to replace a real statistics course or workshop, but to serve as a short and convenient digital refresher for psychology students who have already had an introductory course but need further clarification to tackle *p-*value misinterpretations. Its digital format should enable flexible use as quick practical pre-training before a regular statistics seminar, even in a distance learning setting.

When aiming to develop an effective short-term intervention to tackle misconceptions, refutation texts are a promising starting point^13^. Ideally, refutation texts trigger a cognitive conflict that triggers conceptual change^14^. Various recent short-term interventions based on refutation texts have demonstrated promising effects in tackling misconceptions in the education psychology field, such as the learning-style myth^15–17^. However, these interventions’ material comprised only ~ 200 words and two refutational sentences to address a misconception. It thus seems doubtful, whether an intervention that minimalistic could remedy these notorious *p*-value misinterpretations. Refutation texts alone might not suffice to induce conceptual change^13^, especially considering the tenacity of *p-*value misinterpretations. Falk and Greenbaum^4^ emphasize that pointing out the misinterpretations to students is not enough to overcome this misconception. They gave their learners a critical article to read^8^ with little to no effect. They therefore concluded that conceptual change might be unattainable, “unless strong measures in teaching statistics are taken”^4^. Furthermore, as a recent review paper by Potvin^18^ recommends, it might be rather inefficient for conceptual change interventions to focus solely on a potential cognitive conflict in isolation, without considering the availability of conceptual alternatives and opportunities for a competitive utility evaluation thereof. Therefore, the present paper’s intervention should still be short-term and text-based.

However, its extended refutation text should surpass the typical refutational texts of previous studies by also presenting explanations of the misconceptions and alternative concepts, such as Bayesian statistics. Finally, as Potvin^18^ emphasizes the importance of learners’ processing activities for conceptual change, we aim to assess learners’ mental effort during our intervention.

### Humor to facilitate learning

To enhance an extended refutation text’s potential effectiveness further, the present paper proposes a presentation of funny videos at the start of the intervention. After all, statistics—especially for psychology students—is often associated with negative emotions^19,20^, dread^21^, or even statistical anxiety^22–24^. Field^25^ actually presumes “since time immemorial, social science students have despised statistics (p. XIV).

Hence, to overcome potential negative emotions when dealing with statistics, a little chuckle might be beneficial. In general, laughter and humor have the potential to benefit students’ learning not only by attracting and sustaining attention, but also by creating a more relaxed learning atmosphere and thereby greater student engagement^26^. For instance, Ziv^27^ compared two groups being taught statistics by the same professor, but the professor used humor only in one group. The humor group outperformed the non-humor group in the final exam. Furthermore, prescribed laughter has the potential to not only increase students’ immediate affect but also their creativity, academic efficacy, and well-being^28^. Moreover, learners benefit more from instructors showing positive emotions than when instructors show negative instructors^29,30^. Briefly put, when designing multimedia environments emotions should be considered an important factor^31^ and inducing positive emotions or positive mood has the potential of improving learning^32–35^.

When seeking ways to induce a positive mood during a digital learning environment, short funny videos make sense because of their convenient implementation and their effectiveness in previous research. For example, Nigbur and Ullsperger^36^ used short funny videos (such as cute animals or fail videos) to effectively foster positive mood and awakening. Furthermore, Wang and Chen^37^ found that watching funny videos before learning led to more positive pre-learning emotion than watching neutral videos. However, there was an effect on transfer tests, but none on retention tests.

Against this background, it seems feasible to test whether a prior presentation of short funny videos enhances learners’ mental effort and the intervention’s potential effectiveness.

### Hypotheses

First, the aforementioned high prevalence of *p-*value misconceptions among psychology students should be replicated (Hypothesis 1). However, unlike previous research, this paper does not rely on the usual true-false items but a more valid assessment that combines correctness and subjective certainty. Second, the present short-term intervention that exceeds typical refutation texts should effectively reduce the participants’ *p-*value misconceptions (Hypothesis 2). Furthermore, a prior presentation of funny videos should yield advantages to learners. Against the aforementioned background, it should heighten learner’s mental effort (Hypothesis 3a). The prior presentation of funny videos should also exhibit an additional positive effect on reducing the students’ *p-*value misconceptions, thereby boosting the intervention’s potential effectiveness. (Hypothesis 3b). Finally, and on a side note, the prior presentation of funny videos should also demonstrate an additional positive effect on how the students evaluate the intervention (Hypothesis 3c).

## Method

### Sample and design

One hundred and sixty undergraduate psychology students who had to fulfill part of their research participation requirements were recruited via their university’s platform for research participants. Taking part in the present study required having taken at least one statistical introductory course. The final sample of students who indeed reported having taken such a course and completed the study comprised 157 students (*M*_age_ = 23.36, *SD*_age_ = 4.55; 120 female, 36 male, 1 unknown). The experiment took place completely online and featured a web-based digital learning environment featuring a video phase followed by a text phase.

After providing informed consent, the participants were randomly assigned according to a 2×2-factorial experimental design. The first factor (Factor A) related to the video phase before the text phase, which was either funny (funny condition) or neutral (neutral condition). The second factor (and main factor of interest, Factor B) related to the text phase and featured two conditions: an extended refutation text against *p* value misconceptions (intervention condition) or no such text (control condition). Table 1 shows the experimental conditions and number of participants.

**Table 1:** Experimental conditions and participants.

		Video phase (Factor A)		
		Neutral	Funny	Overall
Text phase (Factor B)	Control	40	39	79
	Intervention	38	40	78
	Overall	78	79	157

### Ethical approval

The study was conducted in accordance with the Ethical Guidelines of the German Association of Psychologists (DGPs). It was approved by the Ethics Committee of Bielefeld University (2024−125). All participants gave informed consent.

### Digital learning environment

#### Video phase (Factor A)

The *funny condition* should heighten the participant’s mood. To achieve this, it featured a series of four video clips (87 s) from YouTube (Bouncing dog, 13 s, IT support hotline, 29 s, trombone on a scooter, 14 s, and rope swing fails, 31 s). The *neutral condition* featured the university’s introduction video (108 s) instead.

A pilot study with 20 undergraduate psychology students who did not participate in the main experiment served the purpose to verify whether these video clips were really considered funny or neutral. The pilot study’s participants rated how funny each video was on a 7-point scale from 1 (*not funny at all*) to 7 (*very funny*). For the funniness rating of the funny condition’s 4-video-series, the mean of the four separate funniness ratings was used. Indeed, the funny condition’s video series was rated as funnier (*M* = 3.78, *SD* = 0.94) than the neutral condition’s video (*M* = 1.6, *SD* = 1.26), *t*(18) = 4.37, *p* < .001, *d* = 2.0 (large effect).

#### Text phase (Factor B)

The *intervention condition* featured an extended refutation text of about 400 words on three pages. Furthermore, the text included explanations and clarifications inspired by and based on the pedagogical concept described by Haller and Kraus^3^. First, it addressed that null hypothesis testing is highly prevalent, but often conducted mindless and ritualistically in social sciences. Next came a short outline of the dispute around null hypothesis testing, namely the hybrid logic, as well as the alternatives, namely confidence intervals, effect sizes and Bayesian statistics. Finally, the third page refuted the common *p-*value misinterpretations via an explanation of what a *p-*value really means—*p*(D|H_0_)—and that you cannot induce *p*(H_0_|D) or *p*(H_1_|D). Furthermore, null hypothesis testing was contrasted to Bayesian testing, which actually enables us to induce the probability of hypotheses given the data. All in all, the extended refutation text had about twice as many the words as standard previous refutations texts used in previous studies^15,17^. Please see the Supplementary Information file 1 for the complete text material.

The *control condition* featured a placeholder, a dummy text so to speak, namely three pages about eLearning that were of similar word count and style than the intervention condition’s text. However, this placeholder text did not address the topic of statistics or hypothesis testing.

### Instruments

#### Demographic questionnaire

Participants filled out a short demographic questionnaire and reported their age, gender, major subject, and statistics courses taken.

#### Misconception score

The misconception score, representing the participant’s *p-*value misconceptions, was assessed as a pre- and posttest before and after the intervention. The questionnaire for that matter relied on the well-established instrument by Oakes^7^, which was also used and adapted by Haller and Kraus^3^. It begins with a short introductory stem (slightly adapted and translated into German): “Imagine the following scenario: You have compared the mean values from an experimental group and a control group (20 participants each) using a *t*-test. H₀ represents the null hypothesis (no difference between the population means). H₁ represents the alternative hypothesis (difference between the population means). You obtain *t* = 2.7, *p* = .01. Please indicate which of the conclusions below you agree with. Please also indicate how confident you are.”

Then came these six statements (slightly adapted and translated into German):

1. You have absolutely disproved H₀.
2. You have found the probability of H₀ is being true.
3. You have absolutely proved H₁.
4. You can deduce the probability of H₁ being true.
5. You know, if you decide to reject H₀, the probability that this decision is wrong.
6. If the experiment were repeated a great number of times, you would obtain a significant result on 99% of occasions.

Unlike with previous versions of this instrument (e.g.^3,7^), participants not only indicated their agreement (*true* or *false*) with each statement, but also their subjective certainty rating from 0 (*very uncertain*) to 4 (*very certain*). A multiplication of the value for correctness (i.e., +1 or −1) with the subjective certainty rating yielded a score ranging from −4 to +4 for each item. The mean for all six items served as the misconception score.

#### Conceptual change score

The conceptual change score was calculated by subtracting the posttest misconception score from the pretest misconception score, reflecting the degree of conceptual change. This procedure was also followed in studies that assessed misconception scores by multiplying correctness and subjective certainty^15,17^. The score ranged from −8 to +8. High positive scores indicate greater conceptual change. For instance, a participant scoring −4 in the pretest and +4 in the posttest would obtain a conceptual change score of 8.

#### Funniness

The participants rated their subjectively perceived funniness of each video on a 7-point scale from 1 (*not funny at all*) to 7 (*very funny*). The mean of the four single funniness ratings served as the funniness rating of the funny condition’s 4-video-series.

#### Mental effort

Mental effort represents the learners’ subjective cognitive engagement while working in the learning environment. This study, like many other studies on multimedia learning environments (e.g.^38–40^), relied on the well-established one item approach, inspired by Paas^41^. More specifically, after the text phase, participants answered the questions “How much mental effort did you invest in studying the text presentation?” on a 9-point rating scale.

#### Evaluation

The participants answered the following four face valid items (translated from German) referring to the intervention’s evaluation on a 6-point scale from 1 (*completely disagree*) to 6 *(completely agree*):

1. I enjoyed working in the learning module.
2. I have a positive overall impression of the learning module.
3. The learning module had the right length in terms of its learning content.
4. I am also interested in similar learning modules.

The mean of these items served as a measure of the participants’ evaluation of the intervention (Cronbach’s α = 0.85).

#### Learning time

The difference between the logged timestamps of when participants started and finished working in the digital learning environment yielded the learning time.

### Procedure

**Fig. 1:**
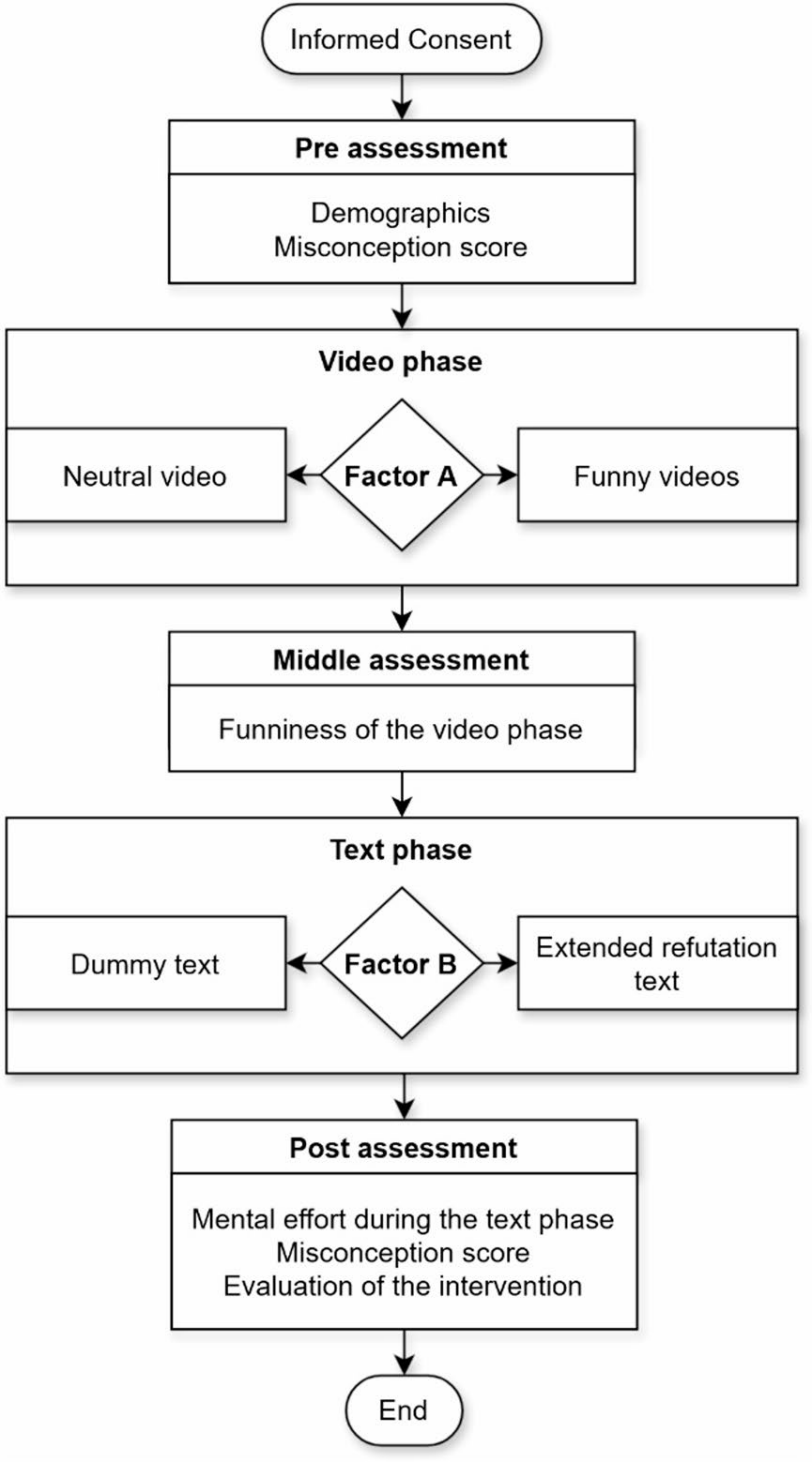
Flowchart of the study design and procedure.

First, the participants filled out a demographic questionnaire and took the pretest. They then worked in one of the four experimental conditions (see Table 1). Finally, they took the posttest. Figure 1 illustrates the study design and procedure in a flowchart.

## Results

The alpha-level was 0.05 and Cohen’s^42^*d* was the effect size measure for *t-*tests (*d* ~ 0.20 small, *d* ~ 0.50 medium, *d* ~ 0.80 large effect). Following recommendations for a direct and efficient way to test specific hypotheses^43^, a-priori contrasts were preferred over of omnibus *F-*tests. Whenever the assumption of homogeneity of variances was violated (checked via Levene’s test), a ‘*t*-test for unequal variances’ with adjusted degrees of freedom was reported^44^. Table 2 shows all the measures.

**Table 2:** Means (and standard deviations) for all measures. ^a^Score from −4 (very low) to +4 (very high), ^b^Score from −8 (very low) to +8 (very high), ^c^Scale from 1 (not at all funny) to 7 (very funny), ^d^Scale from 1 (lowest) to 9 (highest), ^e^Scale from 1 (lowest) to 6 (highest), ^f^Time in minutes.

	Control (*N* = 79)		Intervention (*N* = 78)		
	Neutral (*N* = 40)	Funny (*N* = 39)	Neutral (*N* = 38)	Funny (*N* = 40)	Overall (*N* = 157)
Misconception score: Pretest^a^	0.19 (0.90)	0.25 (1.04)	0.20 (1.11)	0.17 (0.85)	0.20 (0.97)
Misconception score: Posttest^a^	0.27 (0.87)	0.29 (1.10)	1.10 (1.35)	1.49 (1.07)	0.79 (1.22)
Conceptual change score^b^	0.08 (0.47)	0.04 (0.75)	0.90 (1.31)	1.33 (0.88)	0.59 (1.05)
Funniness^c^	1.35 (0.92)	3.66 (1.05)	1.47 (1.01)	3.59 (1.13)	2.53 (1.51)
Mental effort^d^	4.33 (1.83)	5.05 (1.85)	5.82 (1.61)	6.45 (1.62)	5.41 (1.90)
Evaluation^e^	3.18 (1.32)	3.12 (1.06)	3.49 (1.09)	4.00 (1.09)	3.45 (1.19)
Learning time^f^	9.98 (3.45)	10.02 (3.89)	9.98 (4.69)	10.24 (3.62)	10.06 (3.90)

### Preliminary analyses

Several preliminary analyses aimed to check whether the different groups were comparable and the experimental manipulations successful. There were indeed no statistically significant effects of condition on prior misconception score or learning time, *F*(1,153) < 0.05, *p* > .80. Furthermore, participants rated the funny videos as funnier than the neutral video, *t*(153.28) = 13.59, *p* < .001, *d* = 2.17 (*t*-test for unequal variances, large effect). This result indicates a successful manipulation check, demonstrating that the funny videos were indeed funnier than the neutral video.

### Prevalence of *p*-value misinterpretations

The first hypothesis predicted that *p-*value misinterpretations would be very prevalent in the present sample of undergraduate psychology students. Indeed, 98.1% of the participants made a least one mistake in the pretest. The agreement rates for the pretest items ranged from 31.2% (Item 3) to 63.1% (Item 4), making Item 4 the one with the highest difficulty. In other words, 63.1% of the participants believed that the *p* value allows deducing the probability of an experimental hypothesis. However, when looking at the misconception score, which combines correctness and certainty ratings, only 35.7% of the participants had misconception score values below zero. This result indicates a mixture of actual misconceptions and missing concepts. Looking at single items’ scores, 51.0% of the participants had values below zero for the most difficult Item 4. The scores were below zero for Items 4, 5, and 6, which indicates the presence of actual misconceptions, rather than missing concepts. See Table 3 for each item’s scores.

**Table 3:** Percentages of wrong answers (WA) and mean misconception score (MS) with standard deviations in parentheses for each item in the pre- and posttest.

Item	Pretest		Posttest	
	WA	MS	WA	MS
You have absolutely disproved H₀	47.8	0.31 (2.33)	34.4	0.83 (2.43)
You have found the probability of H₀ is being true	33.1	0.91 (1.95)	25.5	1.45 (2.14)
You have absolutely proved H₁	31.2	1.03 (2.27)	17.8	1.62 (2.11)
You can deduce the probability of H₁ being true	63.1	−0.23 (2.00)	38.9	0.89 (2.41)
You know, if you decide to reject H₀, the probability that this decision is wrong	54.8	−0.49 (1.93)	45.9	0.10 (2.19)
If the experiment were repeated a great number of times very often, you would obtain a significant result on 99% of occasions	56.1	−0.33 (2.23)	51.0	−0.17 (2.22)

### Effect of the intervention on conceptual change score

The second hypothesis predicted that the digital intervention built around an extended refutation text would reduce the participants’ misconceptions. The following contrast weights assigned to the experimental conditions were tested: intervention neutral: 1; intervention funny: 1; control neutral: −1; control funny: −1. Testing this contrast yielded a statistically significant effect of the intervention on the conceptual change score, *t*(153) = 7.32, *p* < .001, *d* = 2.34 (large effect).

### Effects of the funny videos

**Fig. 2:**
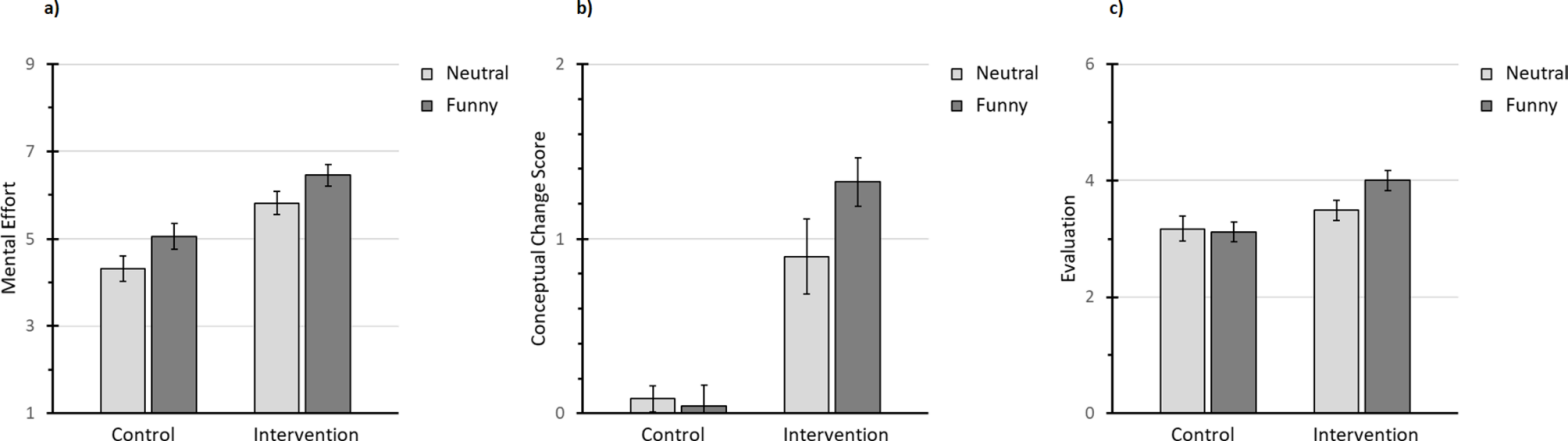
Mental effort (a), conceptual change score (b), and evaluation values (c) in all four experimental conditions, standard error bars.

The third hypothesis predicted that a prior presentation of funny videos would bring advantages to learners. More specifically, Hypothesis 3a predicted an effect of the funny videos (Factor A) on mental effort. A simple *t-*test would suffice to check this. However, if the mental effort assessment is any valid, the extended refutation text (Factor B) should have also induced more mental effort than the placeholder text. Hence, an ANOVA with both Factor A and B as independent variables and mental effort as dependent variable was conducted. The extended refutation text revealed a trivial but large effect on mental effort, *F*(1, 153) = 27.34, *p* < .001, η_p_² = 0.15. Furthermore, and more importantly, the funny videos demonstrated a small effect on mental effort, *F*(1, 153) = 6.06, *p* = .015, η_p_² = 0.04. See Fig. 2a for the mental effort in all four experimental conditions.

Hypothesis 3b predicted an additional positive effect of the funny videos on lowering the participants’ misconceptions. The following contrast weights were tested accordingly: intervention neutral: −1; intervention funny: 1; control neutral: 0; control funny: 0. There was an additional positive effect of the intervention with funny videos compared to the intervention with neutral video that was statistically significant, *t*(153) = 2.09, *p* = .038, *d* = 0.47 (medium effect). Figure 2b illustrates the conceptual change score in all four experimental conditions.

Finally, and more on a side note, Hypothesis 3c predicted an additional positive effect of the funny videos on the participants’ evaluations. Using the same contrast weights as when checking Hypothesis 3b, the intervention with funny videos did indeed show an additional positive effect on participants’ evaluations compared to the intervention with neutral video, *t*(153) = 1.98, *p* = .049, *d* = 0.45 (medium effect). Figure 2c shows the evaluation values in all four experimental conditions.

## Discussion

### Theoretical contributions and practical implications

The present paper offers three innovative contributions to the field of teaching and learning research methods. More specifically, these contributions involve addressing notorious *p-*value misinterpretations among psychology students.

The first contribution is assessing *p-*value misconceptions by combining correctness and subjective certainty. Older research (e.g.^3,4,7^) assessed the prevalence of these misconceptions focusing on answer correctness without considering participants’ certainty. By contrast, the present study did consider and assess participants’ certainty, allowing us to differentiate between missing conceptions and actual misconceptions. Not only did we replicate the well-known prevalence of *p-*value misinterpretation among psychology students (Hypothesis 1) and find as many as 98% of the participants making a least one mistake in the pretest. This finding is in line with Haller and Kraus^3^ who found that 100% of their participating psychology students endorsed at least one misinterpretation. We also gained a more fine-grained picture: A closer look at the misconception scores revealed a mixture of actual misconceptions and missing concepts. For instance, the most difficult item (i.e., “You can deduce the probability of H₁ being true”) was answered incorrectly by most students. More than half of them did so with high subjective certainty. This indicated the presence of an actual misconception, namely that a *p-*value allows deducing an experimental hypothesis’ probability.

It seems plausible that changing learners’ prior conceptions may be more difficult when they are held with high subjective certainty. Ohlsson^45^ suggests that conceptual change can occur when confidence in the contender theory exceeds the confidence in the resident theory. However, as Potvin^18^ argues in a recent review paper, further research is needed due to the complex mechanisms and mixed results regarding learners’ confidence as a preliminary condition for conceptual change. High confidence could indeed make prior conceptions more robust and thus more difficult to change, but it could also induce more meaningful conflicts and thus more changes.

This study’s second contribution focuses on an instructional measure to indeed change learners’ prior conceptions by developing and experimentally testing a highly efficient and innovative digital intervention. This intervention was built around an extended refutation text. Inspired by the pedagogical concept described by Haller and Kraus^3^ and the recommendations by Potvin^18^, it addressed the disputes concerning null hypothesis testing, and explained and refuted misconceptions by contrasting null hypothesis testing to Bayesian testing. The digital intervention had revealed a large positive effect on the participants’ conceptual change score (Hypothesis 2). This finding demonstrates the potential of a short-term digital intervention to reduce students’ notorious *p-*value misconceptions. While being still short-term, its detailed refutations and explanations exceed recent refutation text studies’ material^15–17^.

From a more practical and educational perspective, the purpose of this brief intervention was of course not to replace a real statistics course. Rather, it could serve as a short and convenient digital refresher to help psychology undergraduates who have already passed an introductory statistics course to reduce their *p*-value misconceptions in about 10 min. As a convenient initial step, a pre-training unit so to speak, instructors could hand it out to their students before a regular statistics seminar.

We deliberately tested our intervention as a whole against a no-treatment control condition that did not comprise statistical material. We thereby followed the design of recent instructional psychology studies on short digital interventions that used off-topic or no-treatment control conditions to test their interventions as a whole (e.g., on argumentation knowledge^46^ or misconceptions^47^). This approach seems reasonable from an ecological validity perspective, as psychology students usually attend their courses without any prior digital refresher. Hence, the present study shows the advantage our intervention yields compared to a typical baseline of no intervention. This approach also seems reasonable from a theoretical perspective. Unlike previous studies that compared a refutational text to a standard text, our aim was not to test the effectiveness of the typical two short refutational sentences that the standard text lacks. Rather, we aimed to test the effectiveness of an extended text that also includes explanations of *p*-value misinterpretations and alternative concepts, such as Bayesian statistics.

Nevertheless, given the tenacity of *p-*value misinterpretations and the ineffectiveness of previous treatments, the effectiveness of our intervention compared to a no-intervention control group is not trivial. Rather, it is the result of a carefully designed intervention, built on the pedagogical concept described by Haller and Kraus^3^ and on Potvin’s^18^ conceptual change recommendations. Overall, the present digital intervention, which takes less than 15 min, is a small instructional investment with a significant yield.

As a third and final contribution, this study tackles the challenge of negative attitudes towards statistics, which can make it harder to reduce *p*-value misconceptions. It demonstrates a rather unorthodox humor intervention with funny video clips for extra effectiveness (and chuckles). A short 1.5 min. series of video clips (such as a bouncing dog and rope swing fails) right before the main intervention enhanced students’ mental effort (Hypothesis 3a) and provided an extra effectiveness boost (Hypothesis 3b). As a welcome side effect, these videos also showed a positive effect on the students’ evaluation of the intervention (Hypothesis 3c). These results underscore the positive effects of humor on learning (e.g.^26^), and the potential of funny videos to induce positive mood^36^. From a more practical point of view, watching just 1.5 min of funny videos easily found on YouTube might indeed be a very convenient and economical way to lighten the learning of statistical concepts and reduce statistical misconceptions.

### Limitations and implications for future research

One of the present study’s limitations is its focus on just one statistical topic, namely misinterpretations of *p-*values. Future research should therefore examine whether similar findings can be achieved when addressing other statistical topics prone to misconceptions, such as confidence intervals^5^.

This study’s sample comprised undergraduate psychology students who had taken at least one statistical introductory course. Hence, the generalizability to other sample is obviously limited. Undergraduate students from social sciences other than psychology might still benefit from the intervention though, given they already have basic knowledge about *t-*tests, *p-*values and hypotheses.

Future research might also focus more on mental effort during such an intervention, because of its potential key function for learning outcomes^46^. Prompts helping students to focus on relevant information (e.g.^48^) or to generate self-explanations (e.g.^46^) are promising measures to increase learners’ mental effort. For instance, future studies should compare different versions of the extended refutation texts, each enhanced by different prompts, to further improve effectiveness.

## Electronic supplementary material

Below is the link to the electronic supplementary material.

Supplementary Material 1

## Data Availability

The dataset generated and analyzed during the current study is available from the corresponding author on reasonable request.
