# Supplementary material for: An extended refutation text and funny videos reduce notorious p-value misconceptions
Source: Sci Rep. 2026 May 23;16:16017. doi: 10.1038/s41598-026-53375-w (PMC13198534; doi:10.1038/s41598-026-53375-w)
Supplement: Supplementary file 1 — Supplementary Material 1 [file 41598_2026_53375_MOESM1_ESM.pdf]

## Supplementary Information for:

# An extended refutation text and funny videos reduce notorious *p*-value misconceptions


## Markus H. Hefter[1,*]

[1]Department of Psychology, Bielefeld University, 33615 Bielefeld, Germany
*markus.hefter@uni-bielefeld.de


## Extended Refutation Texts (Translated from German)

**PAGE 1: Difficulties in Understanding Significance Tests**

In the social sciences, it is common research practice to test hypotheses about a population using significance tests based on a sample (“inferential statistics”).

In psychology, virtually all experimental studies currently rely on significance tests for scientific validation.

Unfortunately, the application of significance tests often resembles a ritual. While most students and researchers can perform the formal procedure of a significance test flawlessly, they struggle to understand the statistical concept of significance.

**PAGE 2: Significance Tests are not Scientifically Infallible**

There is still ongoing scientific debate regarding significance tests.

The debate began with disagreements between Fisher (null hypothesis tests) and Pearson and Neyman (the formulation of an alternative hypothesis and the consideration of Type I and Type II errors). Both camps proposed different concepts of statistical inference.

In the 1950s and 1960s, a kind of hybrid logic combining both concepts was taught in many textbooks and courses. If current textbooks mention this dispute at all, they often give the impression that it was clarified and resolved by later research.

To this day, the opposite is true: the mathematical principle of significance tests, their status in science, and their thoughtless use are all subject to criticism!

First, it should be noted that, in addition to significance tests, there are other approaches in inferential statistics, namely:

- Confidence intervals
- Effect sizes
- Bayesian statistics

**PAGE 3: The Most Common Misconception about Significance Tests**

The most common misinterpretation of a significant test result is the belief that it determines the probability of a hypothesis. This misconception is widespread among students and researchers and even appears in some textbooks!

Most students and researchers have collected data and would now simply like to determine the probability of their hypothesis based on this data. However, that is exactly what a significance test cannot do!

A significance test outputs a *p*-value (the exact significance level). However, this *p*-value tells us nothing about the probability of $H_0$ or $H_1$, or about the magnitude of an effect.

In fact, the *p*-value tells us the probability of the data D given that $H_0$ is true, namely $p(D|H_0)$. That is exactly the inverse of what so many people want, namely $p(H_0|D)$.

However, from this probability of the data under the condition that $H_0$ is true, one cannot derive the probability of a hypothesis! From $p(D|H_0)$, one cannot infer $p(H_0|D)$ or $p(H_1|D)$!

Only Bayesian statistics allows us to make statements about the probabilities of hypotheses, which is precisely what is not possible with significance tests.
